# Differential blockade to assess surgical repair by intraoperative active mobilization in knee injuries-Beyond labour analgesia

**DOI:** 10.4103/0019-5049.79903

**Published:** 2011

**Authors:** G Vijay Anand, M Kannan, D Palaramakrishnan

**Affiliations:** Department of Anesthesiology, Tirunelveli Medical College, Tirunelveli, Tamil Nadu, India

**Keywords:** Differential blockade, dislocation of patella, intraoperative active mobilization, labour analgesia, surgical realignment

## Abstract

Motor-sparing selective epidural analgesia has long been practised in the field of labour analgesia. However, the utility of such techniques in other fields remain limited. We present the successful use of a similar technique of differential blockade in a case of quadriceps plasty with realignment of patella for recurrent dislocation of patella. A very low concentration of bupivacaine and fentanyl was used through continous epidural. The adequacy of repair was assessed intraoperatively by active movement of operated limb by patient himself.

## INTRODUCTION

Motor sparing selective epidural analgesia has long been practised in the field of labour analgesia. Such differential blockade techniques have not been taken beyond labour analgesia. We ventured to apply differential blockade in an orthopaedic surgical procedure requiring active movement of the knee joint intra-operatively. We report a unique case which employed differential blockade beyond labour analgesia.

## CASE REPORT

A 21-year-old healthy male was admitted to the orthopaedic department with history of trauma and instability over his left patella. He was diagnosed to have a recurrent dislocation of the patella. The procedure planned was “quadriceps plasty with lateral release and proximal realignment of the patella”. He was then investigated and assessed under ASA I.

The day before surgery, the patient was explained about the nature of the surgical procedure, the mode of anaesthesia and the events he would be expected to co-operate during the procedure. Informed consent was obtained.

We planned a segmental selective epidural analgesia for the procedure. The drug was chosen to be bupivacaine in two dilutions 0.166 and 0.125% with fentanyl 3μg/ml in both the dilutions.

**Figure 1:**
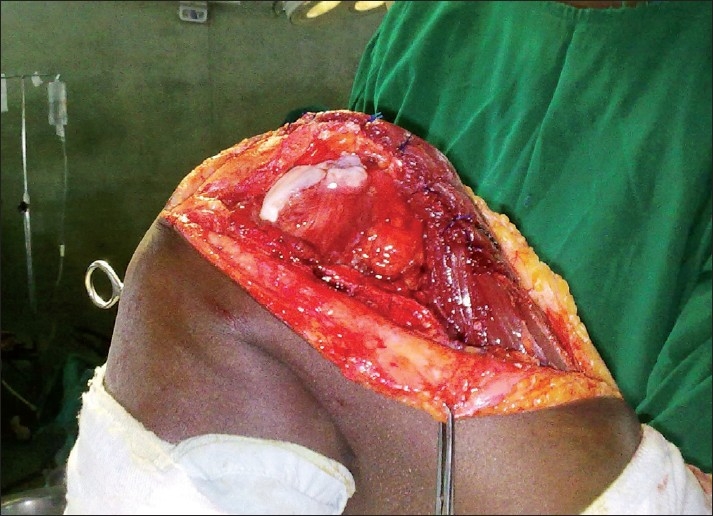
Intra-op mobilization of knee

With a 17G Tuohy epidural needle, the epidural space was approached through L_4_-L_5_ interspace, and identified by loss of resistance to air. A 19G catheter was then threaded and placed 5 cm inside. Standard test dose was not employed, but the initial dose of 12 ml of 0.166% bupivacaine with fentanyl 3 μg/ ml was fractionated into 4 ml aliquots. Fifteen minutes after the injection, sensory blockade was assessed with pin-prick and found to be satisfactory. Motor blockade was assessed with Bromage score and wasscore 3. Tourniquet was then applied to the left thigh. Patient did not complain of any pain or discomfort. Surgeon proceeded with the incision of the skin. A mild sedation with propofol infusion 50 μ/kg/min was maintained. Sixty minutes later, the epidural was topped-up with 6 ml of bupivacaine 0.125% with fentanyl 3 μg/ml. After about 1 hr of the procedure, when the initial sutures were in place for the medial plication, and the wound wide open [Figure 1], the patient was instructed to flex and extend his right knee (non-operated side, taken as control), which he did without any trouble. This was done to assess the degree of sparing of motor blockade. He was then asked to repeat the same to his left limb (operating limb). He managed a 90° active flexion at the left knee and then extended it. The degree of surgical repair was assessed by examining the movement of the patella. When found satisfactory, the surgical team proceeded with closure.

## DISCUSSION

Proximal quadriceps plasty with realignment of the patella is a surgical procedure done to treat recurrent dislocation of the patella.[1] When trying to realign the extensor mechanism, when the trochlea is very shallow?, it is extremely difficult, if not impossible, to judge the proper correction yet avoid overcorrection.

If the patient is co-operative, with epidural and regional techniques, selective epidural analgesia would be extremely helpful. As the motor nerves are spared, the patient can remain awake or under light sedation. The anaesthesiologist can instruct the patient, after several repair sutures are in place. The patient then slowly extends the knee actively from 45° to 0° and back again to test the repair. The surgeon will assess the result immediately, and adjustments made until optimal repair is achieved.

The challenge to the anaesthesiologist lies in balancing between good surgical anaesthesia and motor blockade. The choice has to be made between bupivacaine and ropivacaine, and also the optimum concentration that would give good surgical anaesthesia without motor blockade.

Hector *et al*.,[2] estimated the motor-blocking potencies of bupivacaine and ropivacaine. Ropivacaine was significantly less potent for motor block, at 66% that of bupivacaine. For motor block minimum local analgesic concentration of bupivacaine was 0.326% [95% confidence interval (CI), 0.285-0.367] and for ropivacaine was 0.497% (95% CI, 0.431-0.563) (*P*=0.0008). The ropivacaine/ bupivacaine potency ratio was 0.66 (95% CI, 0.52-0.82).

Owen *et al*.[3] studied that 0.125% ropivacaine is similar to 0.125% bupivacaine for labour analgesia using patient-controlled epidural infusion. They found no significant differences in local anaesthetic use, analgesic characteristics, or side effects between 0.125% ropivacaine and 0.125% bupivacaine and concluded that these two drugs are clinically indistinguishable at this concentration.

Lyons *et al*. have shown that fentanyl 3 μg/ml may be the optimal dose when the aim is bupivacaine sparing extradural analgesia during labour.

As the procedure required a lower concentration and a low volume of local anaesthetic, we decided that bupivacaine would suffice, because ropivacaine did not have any added advantage.[2–7] To ensure adequate surgical anaesthesia, we opted for an initial concentration of 0.166% bupivacaine with fentanyl 3 μg/ml and later to top-up with 0.125% bupivacaine. The initial volume of 12cc achieved good surgical anaesthesia without motor blockade. Onwards the analgesia was maintained with a top-up dose of 6 ml of 0.125% bupivacaine 60 minutes after the initial dose. Mobilization of the operating limb was required 1 hr after when the initial repair sutures were placed. The range of motion exhibited by the patient on the table was satisfactory to the surgeon, when they decided to proceed with closure of the wound.

Similar technique has been described in Chapman’s Text Book of Orthopaedics.[1] But to our knowledge, no report on any such case anaesthetized and operated has been published till date. Hence we publish this case report which was a unique anaesthetic challenge.

## CONCLUSIONS

Motor-sparing selective epidural analgesia was attempted successfully in the surgical realignment of patella, which required active movement of the operating limb to assess the degree of repair. Our endeavour to employ selective analgesia beyond obstetrics did bear a fruit. More studies are needed in this aspect to form an effective protocol.
